# Cerebral Blood Flow Monitoring in High-Risk Fetal and Neonatal Populations

**DOI:** 10.3389/fped.2021.748345

**Published:** 2022-01-11

**Authors:** Rachel L. Leon, Eric B. Ortigoza, Noorjahan Ali, Dimitrios Angelis, Joshua S. Wolovits, Lina F. Chalak

**Affiliations:** ^1^Department of Pediatrics, University of Texas Southwestern Medical Center, Dallas, TX, United States; ^2^Department of Psychiatry, University of Texas Southwestern Medical Center, Dallas, TX, United States

**Keywords:** cerebral autoregulation, cerebroplacental Doppler, fetal MRI, congenital heart disease, hypoxic ischemia encephalopathy (HIE), near-infrared spectroscopy, neonatal brain, fetal brain

## Abstract

Cerebrovascular pressure autoregulation promotes stable cerebral blood flow (CBF) across a range of arterial blood pressures. Cerebral autoregulation (CA) is a developmental process that reaches maturity around term gestation and can be monitored prenatally with both Doppler ultrasound and magnetic resonance imaging (MRI) techniques. Postnatally, there are key advantages and limitations to assessing CA with Doppler ultrasound, MRI, and near-infrared spectroscopy. Here we review these CBF monitoring techniques as well as their application to both fetal and neonatal populations at risk of perturbations in CBF. Specifically, we discuss CBF monitoring in fetuses with intrauterine growth restriction, anemia, congenital heart disease, neonates born preterm and those with hypoxic-ischemic encephalopathy. We conclude the review with insights into the future directions in this field with an emphasis on collaborative science and precision medicine approaches.

## Highlights

- Cerebral autoregulation is a developmental process that can be disrupted in neonates with congenital heart disease, hypoxic-ischemic encephalopathy, and those born preterm.
- Novel methods to assess cerebral autoregulation in these populations can be used to target patient-specific hemodynamic parameters.

## Introduction

**Figure 1:**
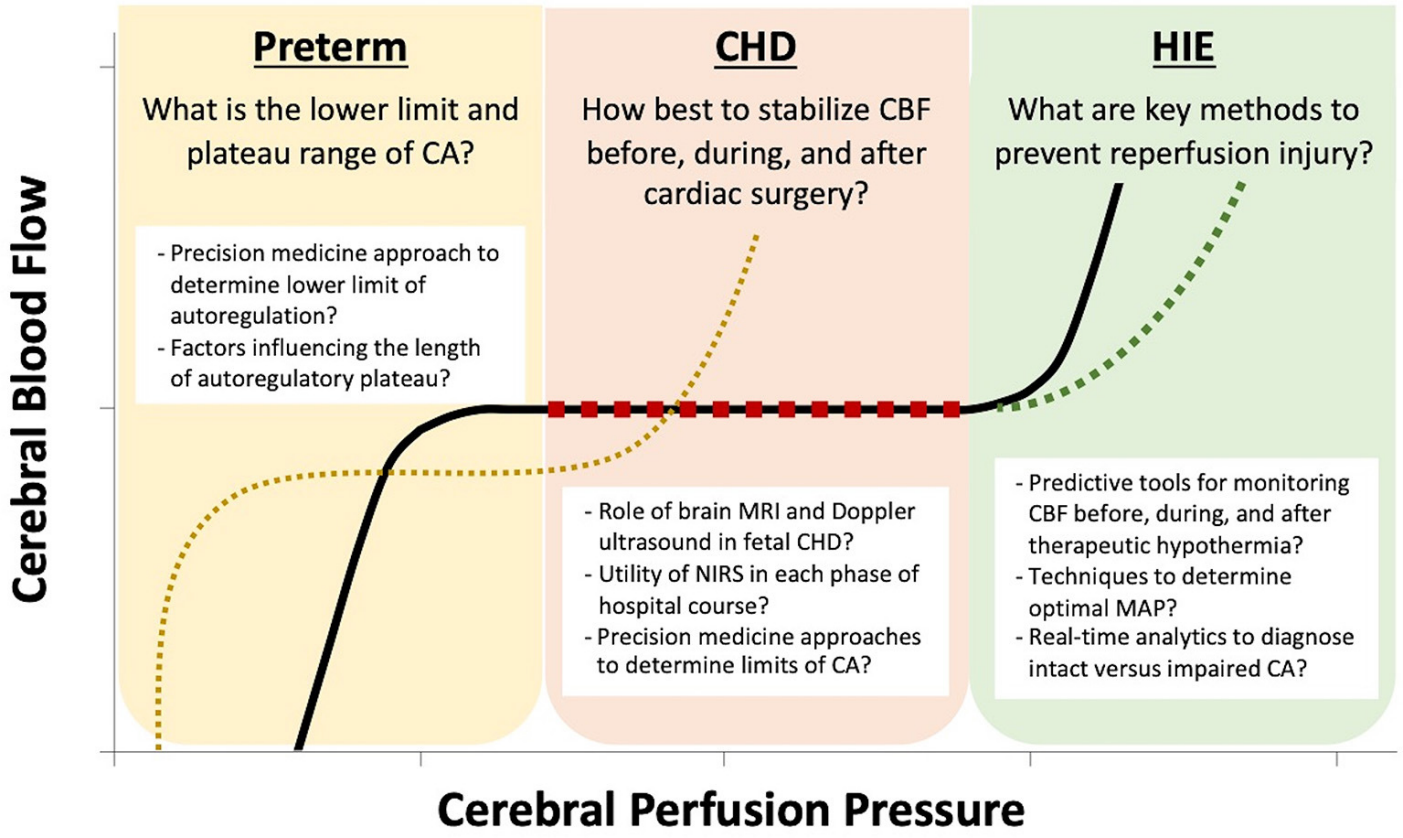
The key questions regarding cerebral autoregulation (CA) are unique to each neonatal pathophysiology. In the preterm neonate, due to immature mechanisms of cerebrovascular adaptation, there is a limited plateau of stable cerebral blood flow (CBF) and there is no strong evidence to guide the optimal lower limit of cerebral perfusion pressure (CPP) by gestational age. In the neonate with congenital heart disease (CHD), postnatal hypoxia and stabilization of CBF before, during, and after cardiac surgery are the prominent challenges. For neonates with hypoxic ischemic encephalopathy (HIE), avoidance of reperfusion injury plays a key role in limiting neurologic damage.

Cerebral autoregulation (CA) is the physiologic adaptation in cerebrovascular resistance across a range of cerebral perfusion pressure (CPP; typically estimated using mean arterial pressure, MAP) in order to promote stable cerebral blood flow (CBF). The maturation and differences in these response mechanisms in fetuses and neonates, particularly those at highest risk for cerebrovascular abnormalities, is poorly understood. Early studies in the chronically instrumented fetal sheep helped shape our current understanding of cerebral hemodynamics in the fetus (1–6). Many of these findings have not yet been confirmed in the human fetus owing to the lack of non-invasive methods other than Doppler ultrasound, which has key limitations, until recent innovations in advanced fetal MRI. This review will explore data on both traditional and novel methods for monitoring CBF and CA in the fetus and neonates with a specific focus on those with pathology that makes them vulnerable to perturbations in CBF (Figure 1).

## Physiology of Cerebral Blood Flow

CBF is highly related to cerebral metabolism and arterial blood gases. Cerebral vasculature is particularly sensitive to the partial pressure of carbon dioxide (P_a_CO_2_), and this reactivity is present in the late-gestation fetus and neonate as well (7). Vascular sensitivity to P_a_CO_2_ occurs throughout the cerebrovasculature, from the major feeding vessels in the neck to the parenchymal and pial arterioles (8). Hypercapnia has the well-known effect of vasodilation while hypocapnia results in cerebrovascular constriction, both of which occur through changes in the local extracellular pH surrounding smooth muscle cells of vessels (9). The sensitivity of the cerebrovasculature to hypoxia is less pronounced and is mediated through adenosine and nitric oxide synthase. Nitric oxide (NO) is a potent vasodilator of cerebrovasculature and is produced by a variety of cell types within the central nervous system, with many studies showing NO-mediated fluctuations in cerebrovascular tone result from not only hypoxia, but multiple stimuli including hypercapnia, cytokines, and activation of the parasympathetic fibers surrounding vessels (10).

Fetal cerebral arteries differ in both structure and function from their adult counterparts (11). The development of the vascular smooth muscle cell layers form around 20–24 weeks gestation and are first evident in vessels that later become the pial arteries (11). The appearance of the muscularis layer of cerebral arteries progresses from pial vessels inwards and concludes around term gestation (12). Pial vessels are traditionally considered the primary site of CBF autoregulation. Capillary-level regulation of CBF may also occur through poorly understood mechanisms involving pericytes (13, 14), which are the cells that make up the blood-brain barrier. Evidence of their potential ability to act as regulators of CBF is mounting. They are known to be contractile, are in direct contact with vascular endothelial cells, and have been shown to migrate alongside the vascular endothelial cells in the developing brain (15).

CBF is known to increase as gestation progresses owing to the increasing metabolic demands (16) and by the third trimester accounts for approximately 25% of combined ventricular output (17). Fetuses have wide minute to minute fluctuations in CBF (4, 18) with a progressive increase in cerebrovascular diastolic flow as pregnancy advances (19). This increase in diastolic flow comes with a slight but steady decline in cerebrovascular resistance that coincides with rapid fetal brain development, but in normal pregnancy remains at a higher resistance than that found in the umbilical arteries throughout gestation (20).

CBF during the transition from fetal to neonatal life is a topic of active investigation, particularly for vulnerable neonatal populations. In the healthy neonate, intact mechanisms of CBF autoregulation ensure steady cerebral oxygenation and glucose delivery at transition. At birth, the low-resistance vascular bed of the placenta is disconnected from systemic circulation while pulmonary vascular resistance falls precipitously with initiation of ventilation. When these events happen concurrently, as expected, the net change in MAP and CBF is minimal. Problems in maintenance of CBF arise when the normal drop in pulmonary vascular resistance does not occur or other factors lead to hemodynamic instability.

Postnatal CBF continues to increase throughout the first 3 years of life with a higher contribution from the posterior circulation compared to adults that peaks in toddlerhood (21). In healthy term infants, CBF is greatest in the brainstem, insula, thalamus and basal ganglia (22). Global CBF estimates in the postnatal period depend greatly on the method used and vary significantly with measurements of 13 ml/100 g/min reported by arterial spin labeling MRI (22), an average of 18 ml/100 g/min by sonographic flowmetry (16), as much as 23 ml/100 g/min by positron emission tomography (23), and approximately 18 ml/100 g/min by near-infrared spectroscopy (NIRS) (24).

## Fetal and Neonatal CBF Monitoring Techniques

CBF is a complex physiologic system with multiple factors that influence its assessment in the evolving maturation from fetal to neonatal life. The techniques used to monitor CBF have also evolved significantly, but each has key benefits and limitations. These techniques are described in detail in the following section and summarized in Table 1.

**Table 1:** A brief description, indication for use, clinical significance, and limitations of key diagnostic studies in newborns at risk for cerebral injury.

**Technique**	**Description/metrics**	**Indication for use**	**Clinical value and characteristics**	**Limitations**
**Fetal assessment tools**				
Doppler ultrasound	MCA PI PI = (PSV–EDV)/TAV	Assess fetal brain blood flow	MCA PI normally has a high value The mean value slowly decreases through gestation (after 28 weeks). A low PI reflects the redistribution of cardiac output to the brain (brain sparing theory)	Low MCA PI in CHD could associate with placental abnormalities and/or cerebral vasodilation (CPR might be better to differentiate this)
	Cerebral/placental ratio (CPR) = MCA PI/UmA PI	Assess fetal blood flow distribution.	Last trimester: High CPR, might relate with impaired placental perfusion and high risk for stillbirth In patients with CHD: CPR <1 was associated with EEG abnormalities and low cognitive scores Values are not affected by fetal heart rate variability. Values assume that the diameter of the vessels remain stable (not dynamic)	In healthy pregnancies, poor prediction of adverse outcomes. Falsely low CPR could result in efforts for early delivery Values affected by variation on technique (e.g., angle of the probe), High Interobserver variability
MRI	Fetal MRI Descriptive brain technique, widely available	Assessment of architecture and volume of brain in CHD.	Delay of brain maturation for fetuses with severe CHD can associate with lower developmental scores.	Moving artifact, not quite standardized for prognostication.
	Advanced MRI techniques Combined cardiac MRI with T2 mapping. MRI-coordinated with ECG	Assess tissue oxygen saturations.	Valuable in assessment of IUGR or Monitoring of infants with cardiac malformation Allows identification of small blood vessels within tissue, quantifies velocity of moving fluids and fetal blood flow	Requires timing of image acquisition with the cardiac cycle (difficult to acquire)
Heart Rate	HR Variability	Autonomic nervous system control of CBF	Lack of HR variability indicative of hypoxia Traditionally used in clinical obstetric assessment during labor New analytics of HR Variability under investigation in postnatal assessment	Non-specific in the fetus Requires continuous monitoring and can be difficult to interpret Research use only in the neonate
**Neonatal assessment tools**				
NIRS	Cerebral tissue saturations, ScO_2_, (by NIRS) are assessed in comparison with MAP, and oxygen and CO_2_ blood content	Assess CBF and autoregulation (when coupled with cerebral perfusion metric such as BP)	ScO_2_: Increases immediately after birth, stabilizes, and slightly decreases in the first 72 h and increases afterwards Use of NIRS, in preterm newborns has been used (as investigation tool) in combination with BP, CO_2_ data for prevention of IVH, etc Clinical significance is questionable and under investigation (e.g., SafeBoosC II trial)	Normal values are not standardized, with wide range (65-90%), which are difficult to interpret Assesses only superficial tissues (cortex in term, cortex and possibly higher white matter layers in preterm) Assumes that oxygen consumption in brain is homogenous For autoregulation assessment need data synchronization software (not widely available)
	Wavelets transform coherence analysis	Dynamic coherence assessment between CBF (cerebral NIRS) and brain electrical activity using aEEG over time using mathematical models (wavelet transform) to assess neurovascular coupling (NVC).	In the normal brain at term, there is temporal and spatial coupling between neuronal activation and blood flow (i.e., NVC) In HIE, there is a well described uncoupling (lack of significant coherence)	Not widely available, experimental. Difficult to interpret routinely.
	Hemoglobin volume phase index	Assesses total Hg values (by NIRS) correlated with BP data.	Correlates with pressure reactivity index (typically requires invasive cerebral monitor), a modality validated in children after TBI Has been used to assess ideal BP after cardiac surgery High Hg Volume index in the first 24 h of life after HIE has been associated with poor long-term outcomes and severe injury by MRI	Although has some prognostic validity, remains experimental Not widely used (only small studies in newborns)
Doppler ultrasound	RI	Assess degree of vasodilation of cerebral arteries	Normative values in healthy term infants established Can be used to predict some outcomes in neonatal HIE Few studies provide information on normative values in other populations such as those born preterm and neonates with CHD	Highly affected by other hemodynamic influences such as presence of PDA, other cardiac shunts, hypotension, etc Influenced by location of assessment (transfontanelle versus transtemporal) Loses predictive validity in HIE during therapeutic hypothermia
	SVC Flow	Indirectly assess CBF by measuring cerebral venous return which comprises 70–80% of SVC flow	Primarily used in preterm populations Predictive of some adverse outcomes such as IVH in preterm neonates	Correlates weakly with cerebral NIRS complicating interpretation Affected by multiple clinical factors—gestational age, hours after birth, PDA diameter, mean airway pressure, etc—thus preventing strong correlation with outcomes in heterogenous patient populations
PET	Xenon-133 clearance	CBF assessment	Detailed data on CBF and fluctuations can be provided	Experimental, not widely available. Invasive

*Of note several of these studies are experimental and not widely used in clinical practices. aEEG, amplitude-integrated electro encephalogram; BP, blood pressure; CBF, cerebral blood flow; EDV, end-diastolic velocity; HIE, hypoxic ischemic encephalopathy; MAP, Mean arterial pressure; MCA, middle cerebral artery; MRI, magnetic resonance imaging; NIRS, Near infrared spectroscopy; PDA, patent ductus arteriosus; PI, pulsatility index; PSV, peak systolic velocity; RI, resistive index; ScO_2_, regional cerebral saturations; SVC, superior vena cava; TAV, time-averaged (mean) velocity*.

### Prenatal Doppler Ultrasound

This relationship between cerebrovascular resistance and resistance in the umbilical arteries provides the basis for *fetal* CBF monitoring using Doppler ultrasound. The cerebral/placental ratio (CPR) is also known as the cerebral/umbilical ratio and is a primary indicator of fetal blood flow distribution (25–30). This metric is a ratio of the relative pulsatility indices (PI) in the cerebral and placental circulations (as measured in the umbilical artery, UmA) defined by the equations,


$$\begin{array}{ll} CPR & =\;\frac{MCA\;PI}{UmA\;PI}\;\text{where}\\ \;PI & =\;\frac{\left(peak\;systolic\;velocity-minimal\;diastolic\;velocity\right)}{mean\;velocity} \end{array}$$

However, limitations to this method of CA monitoring exist. In the third trimester, low fetal CPR is associated with high uterine artery Doppler indices and is thought to be a consequence of impaired placental perfusion that increases the risk for stillbirth (31). However, it may also result from cerebrovascular vasodilation, which is common in fetuses growing poorly and is discussed further in the section on intrauterine growth restriction (IUGR). The use of CPR as a screening tool in otherwise healthy pregnancies has been shown to only weakly predict adverse outcomes and there is concern that overuse of this metric may expose women and neonates to unnecessary interventions such as earlier gestational age delivery (32).

### Postnatal Doppler Ultrasound

Similar to prenatal Doppler ultrasound monitoring of CBF, postnatal measurements can be useful to determine cerebrovascular dilatation as well. The primary metric used postnatally is the resistive index (RI), which is derived from the systolic and diastolic velocities in the cerebral arteries as described,


$$\begin{array}{l} RI=\;\frac{\left(peak\;systolic\;velocity-end\;diastolic\;velocity\right)\;}{peak\;systolic\;velocity} \end{array}$$

Normative values for newborn cerebral artery RI are well defined and typically fall between 0.55 and 0.8 (33, 34). Although RI is not sensitive to the angle of insonation, there are limitations to its use including variations based on location of measurement (e.g., anterior fontanelle or transtemporal) (33), and differences based on technique (35). As such, a recent systematic review concluded that there is no clear evidence to support following RI in preterm infants (36), although RI may still have utility in neonates with hypoxic-ischemic encephalopathy (HIE) (37).

### MRI Techniques

Advanced magnetic resonance imaging (MRI) technologies that contribute to adult research and care are now finding utility in pregnant people and the fetus. Using advanced MR imaging techniques that combine fetal cardiac MRI with T2 mapping to determine tissue oxygen saturations have enhanced our understanding of fetal circulation (38). These experiments effectively replicate in the human fetus, those classic studies in the chronically instrumented pregnant ewe that form the basis for our current understanding of fetal and placental hemodynamics (39–42). Several key differences in the human fetus have been described by Seed and colleagues, who are pioneering the application of these new technologies (43–46).

T2 mapping is an MRI method that allows identification of small blood vessels within tissue. When combined with phase contrast MRI, which quantifies velocity of moving fluids, accurate measurements of fetal blood flow are possible. One major obstacle to this imaging is the necessity of timing image acquisition with the cardiac cycle (47–51). Cardiac gating is used to time data acquisition with specific phases of the cardiac cycle, and MRI-compatible fetal ECG devices are now available (48, 51–55). This technology has now been applied to fetal disease states including IUGR (54) as well as the fetus with cardiac malformations (38).

### Near-Infrared Spectroscopy

Modern NIRS is based on the pioneering work of Professor Frans F. Jöbsis, and since his landmark study in 1977 (56), its use has expanded exponentially. NIRS has the advantage of being non-invasive, inexpensive, and continuous, therefore it is the most commonly used technique to measure regional cerebral saturation (ScO_2_) in neonates despite the lack of large randomized clinical trials to support its clinical impact. The NIRS signal represents the oxygen saturation of Hg in the underlying brain tissue and is derived from the difference in absorption of near-infrared light by the oxygenated and deoxygenated heme groups (57). However, the depth of tissue interrogation can be relatively low and likely only provides information on the cortex (58, 59). Using NIRS to estimate CBF relies on the assumptions that the overwhelming majority of oxygen in blood is bound to hemoglobin, total hemoglobin is proportional to cerebral blood volume, and there is uniform oxygen consumption within the brain. The NIRS ScO_2_ value represents a “weighted average” of arterial, venous, and capillary oxygenation with approximately 75–80% of the signal originating from the venous measurement (60). In general, ScO_2_ values between 65 and 90% are considered normal in healthy term neonates (61, 62).

Only when coupled with a measure of CPP can NIRS impart information about CA. Using NIRS to determine periods of intact and impaired CA requires additional data as well as careful interpretation. Systems that provide time-synchronization of the continuous signals from invasive blood pressure monitors, NIRS, peripheral oxygen saturation, heart rate, and respiratory rate are enhancing the utility of NIRS as a monitor for CA.

Wavelet transform coherence analysis was developed as an alternative tool to Fourier transform to analyze non-stationary signals. Its application to our understanding of neonatal CBF has evolved over the past 10 years (63–65). Due to the non-stationary ScO2 signal provided by NIRS, traditional Fourier transform is inadequate to create a unified analysis across the time-frequency domain without dividing the data into arbitrary “bins” or frequency intervals that require an artificial simplification of the signal into a pseudo-stationary form for analysis. The use of wavelet transform coherence analysis does not require such simplification, but instead allows analysis of signal coherence across a spectrum of the time-frequency domain. The application of wavelet transform coherence analysis has been used extensively for neonates with HIE and is further discussed in that section.

Multiple other analytic techniques utilize NIRS or its component values to determine the correlation between CBF and MAP. The cerebral oximetry index uses the regional cortical oxygen saturation and has been validated in animal models of hypotension (66, 67) followed by clinical trials in pediatric cardiac surgery (68, 69). The pressure reactivity index has been used extensively in traumatic brain injury patients and requires invasive intracranial pressure monitoring, thus, it is not suited for neonates. The hemoglobin volume phase index was developed by Lee et al. using the regional hemoglobin from the NIRS calculation to estimate CBF, and is a non-invasive correlate of the pressure reactivity index (70–72). This method has been used to establish an individualized optimal MAP and time spent below this optimal MAP correlated with adverse outcomes (73). The significant differences in the calculated limits of autoregulation based on each of these methodologies complicates their use in clinical practice and necessitates validation of each methodology in specific populations and disease states.

### Methodologic Limitations of NIRS in the Neonate

Despite their widespread use, no randomized studies to date have systematically investigated potential benefits or risks associated with specific ScO_2_ target ranges or provided validation of these analytical tools in any neonatal population. Normative NIRS signals vary considerably by commercial sensor and by individual patient physiology. Only recently investigators have reported on the mathematical conversions to allow comparison of NIRS data between devices (74, 75). Due to these limitations, the European Society of Paediatric and Neonatal Intensive Care recommends against routine clinical use of NIRS in all children with hemodynamic instability and there is no agreement from the group on the significance of a decline in ScO_2_ from baseline or a lower limit of normal (76).

### Other Monitoring Techniques

Early studies on CBF in neonates used a modified xenon-133 clearance method that relied on external scintillation detectors (77–79). These earlier discoveries related to the influence of Hg, hypoglycemia, and blood pressure on neonatal CBF have informed much of the contemporary investigations into neonatal CBF homeostasis. Recently developed methods to estimate CBF based on heart rate variability show promise since invasive monitoring of MAP is not always possible or desirable in all neonates (80). Heart rate variability refers to the variation in time intervals between each heartbeat and is controlled by the autonomic nervous system. This variability is affected by hypoxia and other conditions that compromise CBF, thus making the metric a possible predictor of disturbed CBF (81, 82). One key limitation to using heart rate variability as a clinical tool is correlating it with other physiologic parameters of cerebral oxygenation and the real-time data analysis required for it to be useful (83).

Another method of CBF monitoring is superior vena cava (SVC) flow which is based on the physiologic principle that cerebral venous return contributes as much as 70–80% to total SVC flow. This technique has been applied in preterm populations and may correlate with adverse outcomes (84), although it is subject to influence by multiple factors. In one study, low SVC flow was associated with lower gestational age, higher upper body vascular resistance, larger diameter patent ductus arteriosus (PDA) shunts, and higher mean airway pressure (85). Together, these variables can make interpretation of SVC flow difficult. In addition, SVC flow only correlates weakly with estimations of CBF by cerebral NIRS (86), adding complexity to its use. With further refinement this technique may contribute to the overall assessment of CBF, and is the subject of ongoing investigations to determine its predictive validity on long-term outcomes in preterm neonates (87).

## Cerebral Blood Flow in Vulnerable Fetal and Neonatal Populations

### Intrauterine Growth Restriction

CPR is used in pregnancies complicated by IUGR to quantify the redistribution of cardiac output with increased flow to the fetal brain (traditionally called a “brain-sparing” effect) (88, 89). However, this term may be a misnomer. In a secondary analysis of the large multicenter Prospective Observational Trial to Optimize Pediatric Health in IUGR (PORTO) Study, investigators showed that this redistribution of blood flow to the brain is associated with adverse perinatal outcomes including intraventricular hemorrhage, periventricular leukomalacia, HIE, necrotizing enterocolitis, bronchopulmonary dysplasia, sepsis, and death (90). In fact, fetuses with IUGR and CPR <1 had poorer neurodevelopmental outcomes at 3 years compared to pregnancies with abnormal umbilical artery Doppler alone in the follow up report of 375 patients from the PORTO Study (91). These studies clearly demonstrate that cerebral vasodilation does not result in normalization of brain development.

The cause of this hemodynamic disturbance is most frequently related to placental insufficiency and can lead to deleterious alterations in vasoreactivity of cerebral vasculature when prolonged (92). In fact, IUGR fetuses with normalization of MCA-PI after prolonged vasodilation are at increased risk for fetal demise (93). Postnatally, neonates who experienced IUGR have persistent vasodilation of cerebral vasculature (94). This can result in higher ScO_2_ values by NIRS likely due to a combination of increased cerebral oxygen delivery and a decreased cerebral tissue oxygen consumption (95). The key factor in using this technique is to not rely soley on the single timepoint measurement; rather, a trend in CPR is required to determine where on the trajectory a fetus is in regard to CA.

### Fetal Anemia

A fetus is typically evaluated for fetal anemia (defined as Hg levels less than mean for gestational age (GA) (96) or multiples of the median (MoM) for GA) (97–99) when it is noted to have abnormal fluid collections (ascites, pericardial effusion, hydrothorax, and/or skin edema) or reduced movements. The gold standard for the diagnosis of fetal anemia is fetal blood sampling (FBS), a procedure that imparts a high risk for fetal loss, particularly in hydropic fetuses (100). Doppler ultrasonography can detect fetal anemia based on an increase in the peak velocity of systolic blood flow in the MCA (MCA-PSV), mitigating the risk of fetal loss (97, 100). This non-invasive metric relies on the fetal cerebral autoregulatory capacity to increase CBF in the setting of hypoxia, similar to that seen in fetal IUGR.

The basic principle used by Doppler for the diagnosis of fetal anemia, relates to the observed increase in blood velocity which stems from the increased fetal cardiac output. Due to relative maintenance of cardiac output for small changes in Hg, there is no strong correlation between MCA-PSV and fetal Hg within the normal range. If significant Hg decreases occur, however, the MCA-PSV increases and can be used to determine the Hg value with a fair degree of accuracy (100). An MCA-PSV > 1.5 MoM is used as a cut-off for severely anemic fetuses. In non-hydropic fetuses, sensitivity of a single measurement of MCA-PSV varies between 75 and 95%, with about 10% of false-positive results, and this increases if the study is performed after 35 weeks GA (101). Use of the multiple time points of MCA-PSV can decrease the false positive values (101). As such, for high-risk fetuses, MCA-PSV are typically performed at regular intervals depending on risk assessment. Fetuses with anemia are at risk for multiple complications and may benefit from intrauterine transfusions, underscoring the importance of assessment at regular intervals in those deemed high-risk.

### Infants Born Preterm

Although much has been learned about CBF in preterm neonates, many questions remain including how variations in CBF are related to neurologic pathologies, and the role of various CBF monitoring techniques, particularly in the setting of disturbed hemodynamics such as hypotension, PDA, anemia, and changes in respiratory status. In all neonates, immediately following birth there is an initial increase in CBF followed by a slight downward trend until around 72 h of life. This rise in CBF after birth is more pronounced in preterm infants (102, 103). Immaturity of the autonomic nervous system, especially the parasympathetic control of CBF is particularly underdeveloped in preterm infants and is the presumed cause of these differences between term and preterm infants (104–106). Loss of this autonomic regulation dampens the adaptive cerebral reactivity, making preterm infants susceptible to passive cerebral circulation and putting them at risk for low CBF in the setting of hypotension (107, 108). When accompanied by PDA, this further compromises CBF and the ability to maintain intact CA (109, 110).

Respiratory status changes also affect CBF more acutely in the preterm neonate as they are prone to rapid fluctuations in P_a_CO_2_ after birth due to respiratory distress syndrome and surfactant administration. In addition, fluctuations in MAP acutely impact cerebrovascular responsiveness to P_a_CO_2_ (111). Together, these circumstances put preterm infants at increased risk of impaired CA. Dysfunction of CA has been proposed as the mechanism for preterm brain injuries such as IVH and leukomalacia (112–115). The correlation between NIRS metrics of intact vs. impaired CA and subsequent IVH was recently demonstrated in a cohort of preterm infants studied in the first 4 days of life (116). Infants who developed IVH or died showed a loss of cerebral reactivity with fluctuation in blood pressures, resulting in both hypoperfusion and reperfusion brain injury (116).

Several studies to date have asked the question of whether routine NIRS monitoring in preterm neonates improves outcomes. There is no consensus as researchers from several centers have reported opposing results (117–120). Comparisons between such studies are difficult, however, and differences are likely due to contrasting approaches in the *management* of preterm neonates based on S_c_O_2_ measures. Initial results in the SafeBoosC II randomized controlled trial of NIRS in neonates born extremely preterm did not show an impact on EEG outcomes (121) or neurodevelopmental outcome at age 2 years (119), but an additional Phase III randomized-controlled trial is underway (122). A practical guide to the use of NIRS in neonates was recently published by Vesoulis et al. and the authors conclude that there is a need for large studies of comparative approaches (123), which the SafeBoosC III trial will be a strong contributor. These studies must occur while equipoise still exists.

### Congenital Heart Disease

#### Fetal CHD

In fetuses with CHD, there is mounting evidence that brain development is disrupted prenatally with lagging brain growth (38, 124, 125) and estimated growth delay of ~1 month (126–128). This observation has spurred significant interest in CBF monitoring in this population. The use of CPR presents unique challenges to interpretation in fetal CHD. In many forms of CHD, there is adaptive cerebral vasodilation attributable in some cardiac lesions to a relative fetal cerebral hypoxia. In a small study of fetuses with hypoplastic left heart syndrome (HLHS), dextro-transposition of the great arteries (D-TGA), and tetralogy of Fallot (TOF), CPR <1 was associated with reduced neural synchrony as measured by EEG (129). In addition, in those with CHD and CPR <1, cognitive scores on the Bayley Scales of Infant Development were lower at 18-months of age. The adaptive cerebral vasodilation in the fetus with CHD results in a lower MCA PI, which may or may not be accompanied by placental insufficiency (130–134). Higher rates of placental pathologies are known to occur in pregnancies complicated by fetal CHD (135–139), therefore CPR requires careful interpretation in fetuses with CHD.

#### Postnatal CBF in CHD—Early Adoption of NIRS

NIRS is now commonplace in most pediatric cardiac intensive care units despite the lack of specific normative parameters in neonatal CHD or randomized controlled trials to support its use. Small studies showing improved metrics of care with NIRS use, such as reduction in days on the ventilator in post-operative neonates with CHD (140) have contributed to widespread adoption of the technology. The interpretation of the NIRS signal in the setting of altered CBF in specific CHD physiologies presents unique challenges. In neonates with single-ventricle CHD, ScO_2_ is lower than healthy term infants at baseline [68.0 ± 9.7 (*n* = 28) compared to 80.6 ± 7.9, *n* = 16 in healthy controls; *p* < 0.001] (141), but depending on whether there is right or left-sided obstruction, ScO_2_ values can vary (142). Definitive data on whether NIRS monitoring improves patient care is a topic of active investigation (143–147), although the optimal time to answer this question has likely passed given the near-universal use of NIRS in neonates with operative CHD in the United States.

### Hypoxic-Ischemic Encephalopathy

There is a sense of urgency to improve our understanding of CA in neonates with HIE as their fluctuations in CBF and maintenance of adequate CPP have been shown to correlate with secondary injury (148–154). Avoidance of post-hypoxic reperfusion injury is a key area of concern in these infants (155). Doppler assessment of cerebral RI with measurements < 0.55 indicate loss of CA and correlate with worse outcomes (156, 157). However, in the setting of active therapeutic hypothermia, RI is somewhat less predictive (positive predictive outcome 60%, negative predictive outcome, NPV 78%) (158), but in late cooling RI < 0.55 has a NPV of 86%, which increases to 89% after rewarming (159).

**Figure 2:**
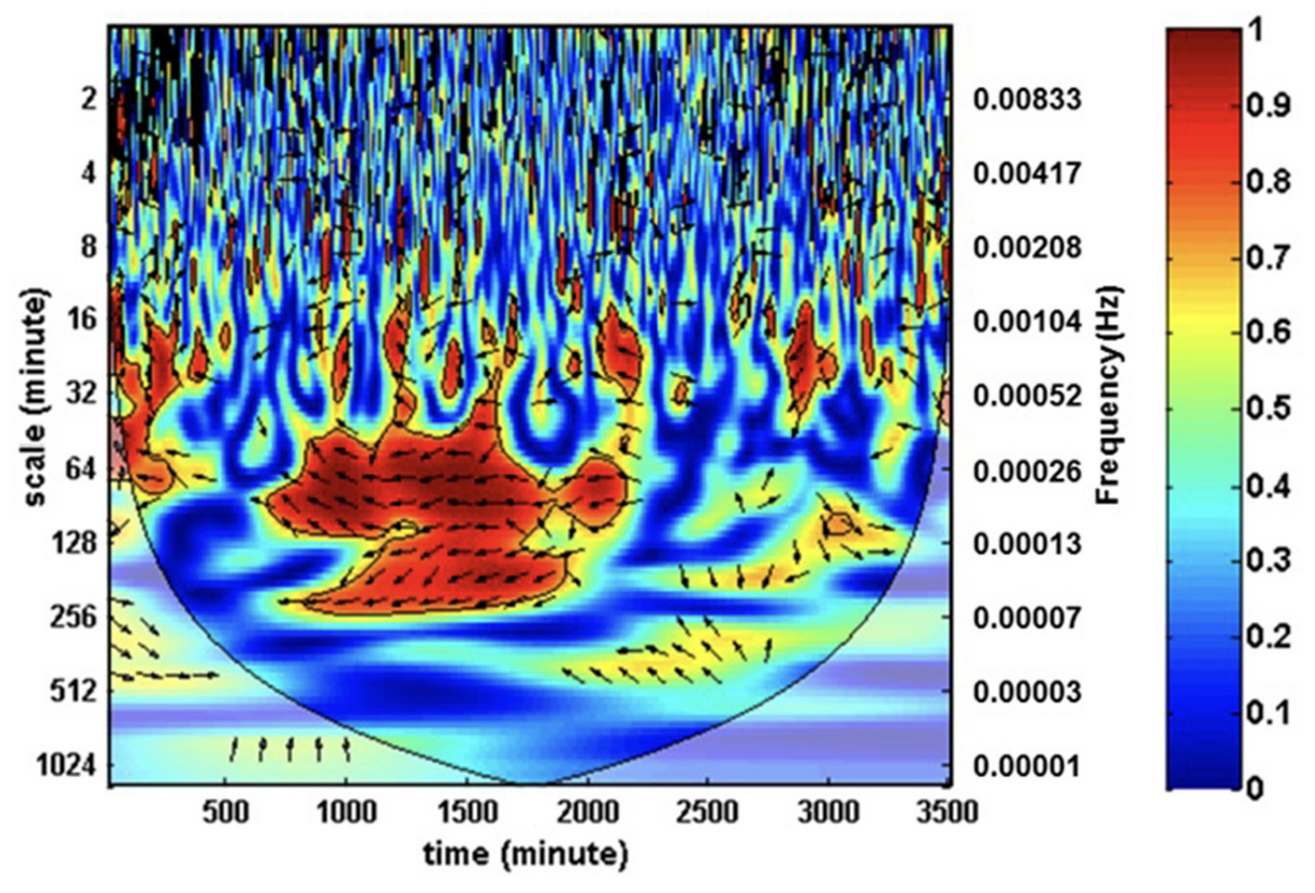
Wavelet transform coherence analysis can be used to characterize cross-correlations between mean arterial pressure and ScO_2_ as a function of a wide range of both time and frequencies. The coherence depicted by color mapping represents the correlations across the time-frequency axis and can be translated into percent coherence from 0 (least coherent, blue) to 1 (most coherent, red). In-phase coherence indicates impaired cerebral autoregulation.

Many studies underway are using NIRS coupled with invasive MAP monitoring to determine the upper limits of CA in asphyxiated neonates in order to develop individualized optimal MAP parameters and improve long-term prognostication (151, 160). With adjuvant therapies on the horizon, this will enhance the impact of therapeutic hypothermia (or replace its use in those patients in whom it is contraindicated) (161–165). Wavelet transform coherence analysis is being used to differentiate those neonates labeled as mild HIE who may benefit from therapeutic hypothermia based on their inability to maintain intact CA (Figure 2). Chalak et al. has further developed the wavelet coherence analysis to quantify neurovascular coupling by analyzing the relation between regional changes in aEEG signals and MAP. This analysis may provide new avenues for classification of HIE injury that will promote a more targeted therapeutic approach, particularly due to the heterogeneity of the HIE diagnosis. Currently, these techniques are limited by the time and computationally intensive analysis required. Therefore, further development of these bedside time-synchronized hemodynamic monitoring tools to include real-time analytics for neonates with HIE is of utmost importance.

Hemoglobin volume phase index, which relies on an estimated relative total hemoglobin from NIRS along with continuous MAP to assess CA, was recently used in a cohort of neonates with HIE. Those with higher hemoglobin volume phase values at 24 h of life (indicating impaired CA) had a higher rate of death or severe brain injury by MRI (166). The author's proposed cutoff of hemoglobin volume phase index had a sensitivity of 86% and specificity of 74% for death or severe brain injury by MRI. This type of early prognostication tools may facilitate the use of adjuvant therapies in specific HIE sub-populations and/or allow for improved decision-making about goal-directed care at a much earlier timepoint than standard treatments currently allow. Next steps should include larger scale multicenter prospective clinical trials to determine the generalizability of this metric.

## Future Directions in CBF Monitoring

### In the Fetus

CBF monitoring of the fetus will be improved significantly through advances in MRI technologies. Currently, the research scans that show the most promise for fetal and placental perfusion are limited to specific institutions that have the expertise required for these customized sequences and their interpretation. Image data sharing between institutions and countries is a major barrier. Commercialization of these MRI scanning sequences would improve their accessibility and data sharing capabilities. The development of artificial intelligence for automated region of interest assignment will be key to improve the pipeline of discovery as will user-friendly interfaces for analysis. These technologies will not replace the inexpensive and readily-available method of Doppler ultrasound for CBF monitoring anytime soon, thus studies to maximize our knowledge and utilization of this tool are also warranted. The CEPRA study (Dutch Trial Registry NTR trial NL7557) is an example of how the fetal Doppler CBF monitoring field is moving forward, the goal of which is to determine the utility of CPR as an indicator for immediate delivery in pregnant people reporting loss of fetal movement (167). These types of multicenter studies are essential to have an impact on clinical practice of fetal monitoring technologies that have already been studied for many years. In contrast, studies of CBF using fetal MRI are in their infancy and require robust sharing of data and technical expertise between centers to move the field forward.

### In the Neonate

For postnatal CBF monitoring, NIRS dominates most recent research and will likely continue to be a favored technology in the neonate. However, in order to move forward with the widespread use of NIRS and its real-time analysis with other hemodynamic monitoring signals to estimate CA, significant investment is required to perform multicenter randomized controlled trials validating its ability to improve outcomes in specific neonatal populations. In neonates with CHD, equipoise is likely lost for clinical studies of NIRS since it is already widely used in these patients in cardiac intensive care units and its benefit in that population has been demonstrated in small studies. Clinical trials of NIRS and time-synchronized analytical tools are being studied in other populations, however, such as neonates with HIE (64, 65, 168) and those born preterm (121, 122, 169).

In addition, the use of NIRS to estimate the upper and lower limits of CA in vulnerable neonatal populations requires additional study and a focus on a precision-medicine approach, which is underway in ongoing clinical trials (170). The optimal MAP to maintain stable CBF are unique to individuals and highly related to their specific pathology and their hemodynamic adaptations (160). In the fetal and preterm neonatal populations the ability to autoregulate CBF is not yet fully mature. For those with CHD, the limits of CA are highly related to the degree of somatic and cerebral hypoxia that drives minute to minute changes in CBF. In each of these cases, uniform thresholds of what is considered adequate MAP are not able to provide the type of precision medicine that is needed to improve patient outcomes. Instead, new tools to analyze and integrate the multitude of data points that clinicians are tasked with interpreting are essential to allow a precision-medicine approach and move the field forward.

Technologies that integrate multiple monitoring modalities into a time synchronized visual representation will allow a new level of interpretation and improve our individualized approach to care. Specifically, in terms of CA, an accurate assessment of upper and lower limits of CA must be made for each patient in order to precisely titrate medications and interventions. Next-level monitoring tools are required to facilitate the rapid recognition of impending hemodynamic instability. Likewise, these predictive analytics will be useful to encourage faster weaning from vasoactive medications, mechanical ventilators, and other treatments. As with all technologic advances in patient monitoring, the question of whether these analytics of the future make a positive impact on patient care must be asked and carefully investigated.

## Conclusions

Traditional methods of CBF monitoring facilitated our foundational understanding of the maturational process of these complex mechanisms and their behavior in the setting of perturbed hemodynamics. However, the use of animal models and reliance on technologies with significant limitations led to a period of stalled progress in the field. Recently developed techniques including those involving advanced MRI, NIRS, and novel statistical methods have sparked renewed progress. These technologies have led to a new understanding of CA as a dynamic process with limitations that are unique to not only a specific pathology, but also unique to individual patients. Progress in this field will hinge upon our ability to integrate the continuously acquired plethora of patient data into clinically useful and actionable decision-making algorithms.

## Author Contributions

RL: concept, drafting, revising, and final approval. EO, NA, DA, and JW: drafting, revising, and final approval. LC: concept, revising, and final approval. All authors contributed to the article and approved the submitted version.

## Funding

This work was supported by National Institute of Health (No. 1R01NS102617) to LC.

## Conflict of Interest

The authors declare that the research was conducted in the absence of any commercial or financial relationships that could be construed as a potential conflict of interest.

## Publisher's Note

All claims expressed in this article are solely those of the authors and do not necessarily represent those of their affiliated organizations, or those of the publisher, the editors and the reviewers. Any product that may be evaluated in this article, or claim that may be made by its manufacturer, is not guaranteed or endorsed by the publisher.
